# Amphotropic murine leukaemia virus envelope protein is associated with cholesterol-rich microdomains

**DOI:** 10.1186/1743-422X-2-36

**Published:** 2005-04-19

**Authors:** Christiane Beer, Lene Pedersen, Manfred Wirth

**Affiliations:** 1 Molecular Biotechnology, German Research Centre for Biotechnology, GBF, Mascheroder Weg 1, D-38124 Braunschweig, Germany; 2 Institute of Clinical Medicine and Department of Molecular Biology, University of Aarhus, Aarhus, Denmark

## Abstract

**Background:**

Cholesterol-rich microdomains like lipid rafts were recently identified as regions within the plasma membrane, which play an important role in the assembly and budding of different viruses, e.g., measles virus and human immunodeficiency virus. For these viruses association of newly synthesized viral proteins with lipid rafts has been shown.

**Results:**

Here we provide evidence for the association of the envelope protein (Env) of the 4070A isolate of amphotropic murine leukaemia virus (A-MLV) with lipid rafts. Using density gradient centrifugation and immunocytochemical analyses, we show that Env co-localizes with cholesterol, ganglioside GM1 and caveolin-1 in these specific regions of the plasma membrane.

**Conclusions:**

These results show that a large amount of A-MLV Env is associated with lipid rafts and suggest that cholesterol-rich microdomains are used as portals for the exit of A-MLV.

## Background

Cholesterol-rich microdomains like rafts and caveolae are specialized regions of the plasma membrane and play an important role for several cellular processes e.g., signal transduction, and for the life cycle of certain viruses (e.g., the entry and exit steps). These domains are enriched in cholesterol, sphingomyelin, ganglioside GM1 and caveolin proteins [1]. The cholesterol molecules are intercalated between the lipid acyl chains and cause a decrease of the fluidity of these membrane regions leading to their resistance against treatment with non-ionic detergents like Triton X-100 at 4°C [1]; therefore, these regions are also referred to as detergent resistant microdomains (DRMs). The specific lipid composition of DRMs leads to the selective incorporation and concentration of specific cellular proteins (reviewed in [1]).

Recently, the envelope protein (Env) of the ecotropic murine leukaemia virus (E-MLV) as well as of human immunodeficiency virus type 1 (HIV-1) were shown to associate with DRMs after transport to the plasma membrane [2,3]. Similarly, Gag proteins of HIV-1 prefer DRMs as cellular destinations after synthesis in the cytoplasm [4-6]. As HIV-1 and E-MLV bud from plasma membrane regions where the viral capsid and envelope proteins are enriched [7,8] the DRM-association of the viral proteins led directly to the idea that DRMs are platforms for assembly and budding (reviewed in [9]).

Glycosyl phosphatidylinositol (gpi) anchoring and fatty acylation have been shown to direct proteins to lipid rafts (reviewed in [10,11]). Mutation of HIV-1 Env or E-MLV Env palmitoylation sites [2,3] or the HIV-1 Gag myristoylation site [4] impaired the association of these proteins with DRMs. Furthermore, knock out of Env palmitoylation sites led to a decreased viral titer due to a reduced Env incorporation into the viral particles [3].

Viral budding from DRMs should lead to a viral membrane composition, which resembles the lipid composition of DRMs and differs from the average distribution of lipids in the plasma membrane [9]. For example, the enrichment of the membrane of HIV-1 with sphingomyelin and cholesterol [12,13] strongly supports a role for DRMs in HIV-1 budding (reviewed in [9]). In a recent report, we showed that a 1.4 fold increase of the cholesterol content of the plasma membrane of NIH3T3 cells resulted in a more than 3-fold increase of viral membrane cholesterol of amphotropic MLV (A-MLV) released from these cells [14]. We suggested that this phenomenon could be due to the involvement of DRMs in assembly and budding of A-MLV. To address this issue, we have here performed density gradient centrifugation, immunocytochemical staining and co-localization experiments using A-MLV Env expressing NIH3T3 and 293 cells.

## Results

### Triton X-100 insolubility of A-MLV Env

To investigate the association of A-MLV Env with DRMs via density gradient centrifugation, 293T cells were transiently transfected with a pHIT-derived plasmid encoding the A-MLV envelope protein [15]. Moreover, expression plasmids encoding enhanced green fluorescent protein (eGFP) (pEGFP-N1, Clontech) were transiently transfected in 293T cells and used as non-DRM marker. Forty-eight hours after transfection, the cells were treated for 10 minutes with 1% TX-100 at 4°C and the resulting cell lysates were loaded on discontinuous density gradients. Due to the insolubility of DRMs, these membrane regions as well as their associated proteins float to the top of the gradient [16].

**Figure 1:**
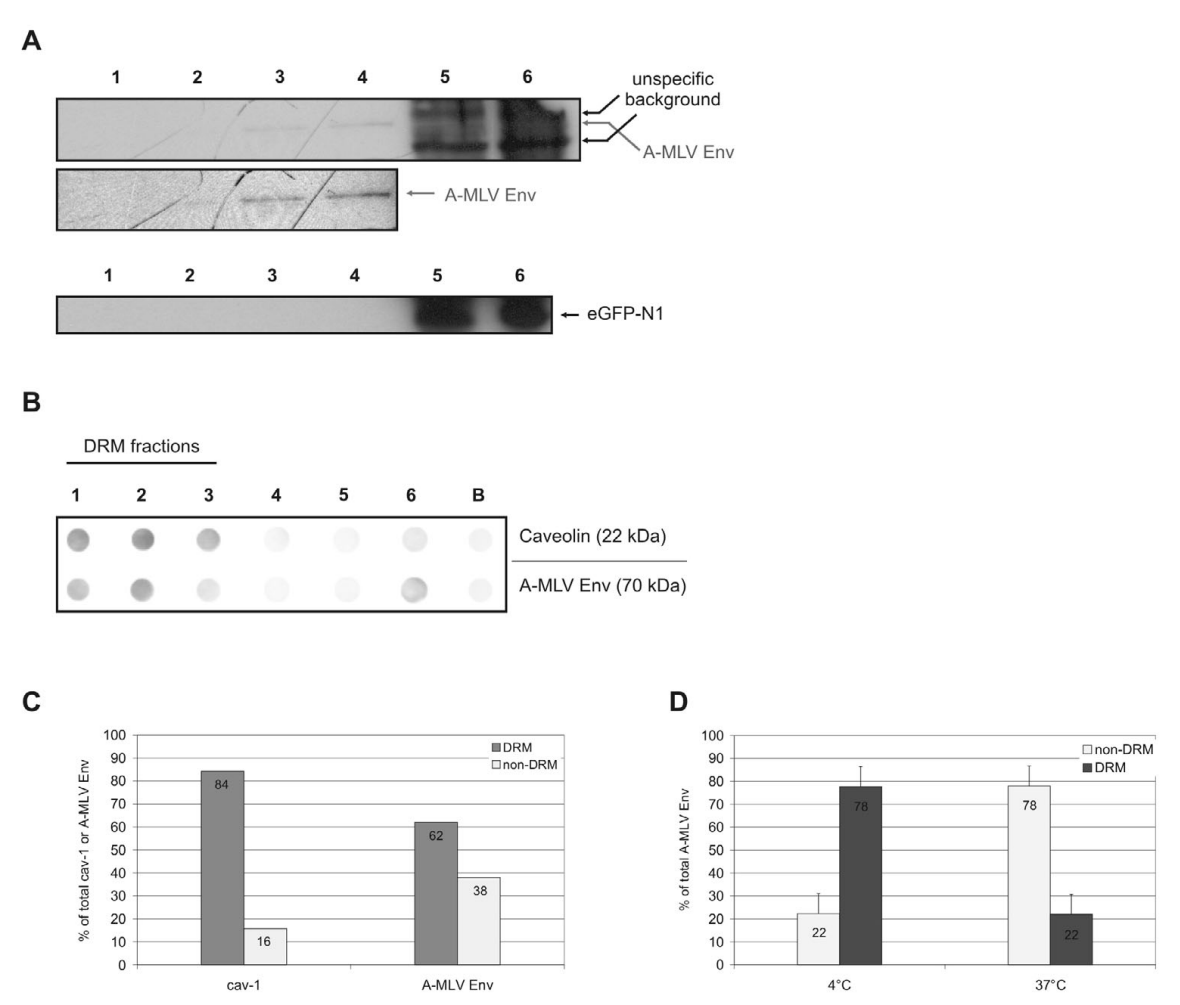
**A-MLV envelope protein associates with detergent resistant microdomains (DRMs). A) **293T cells producing A-MLV were treated with TX-100 at 4°C and loaded on a discontinuous sucrose gradient. Western blot analyses were performed. Fraction 1 corresponds to the top and fraction 6 corresponds to the bottom of the tube. Fractions 1 to 4 contain the DRMs, fractions 5 to 6 the non-DRM membrane fractions. A-MLV Env is found predominantly in the DRM fractions 2, 3 and 4. EGFP, which is localized in the cytoplasm, remains in the soluble fractions 5 and 6. **B) **NIH3T3 cells releasing A-MLV were treated with TX-100 at 4°C and loaded on a discontinuous sucrose gradient. Dot blot analyses were performed. Fraction 1 corresponds to the top and fraction 6 corresponds to the bottom of the tube, respectively. B is the background of the dot blot. Fractions were processed in parallel for immunological detection of cav-1 and A-MLV Env. **C) **Quantification of the dot blot shown in B) using image analysing software. The amounts shown are determined as percentages of the total of all dots; DRM (fractions 1 to 3), non-DRM (fractions 4 to 6). **D) **Detergent soluble supernatant (non-DRM) and insoluble pellet (DRM) of A-MLV producing NIH3T3 cells treated with TX-100 at 4°C or 37°C were investigated for the amount of envelope protein using dot blot analysis. The results of two independent experiments are shown. The amounts shown are determined as percentages of the total of all dots.

Confirming the routine of the fractionation experiments, unmodified eGFP, which is localized in the cytoplasm, was exclusively found in the soluble fractions 5 and 6 (Fig 1A). Therefore, these fractions were considered as detergent soluble fractions. A-MLV Env floated predominantly to the DRM fractions 2, 3, and 4. Fractions 5 and 6 contained high background signals, in which no A-MLV Env specific band could be detected (Fig 1A).

Additional fractionation experiments were performed using A-MLV producing NIH3T3 cells. Analysis of the resulting Dot Blots revealed that at least 60% of the viral Env protein was localized within the detergent insoluble fractions when the cells were treated with TX-100 at 4°C (Fig 1B and 1C). As unspecific background was found only in detergent soluble fractions (Fig 1A), an overestimation of the amount of DRM associated A-MLV Env is unlikely. In addition, TX-100 treatment of the cells at 37°C dissolved rafts and drastically reduced the percentage of Env associated with the detergent insoluble fractions (Fig 1D). In summary, these data imply that a large fraction of A-MLV Env is localized in DRMs.

### A-MLV Env exhibits properties of DRM-associated proteins

To verify A-MLV Env association with DRMs at the cell level, a set of immunocytochemical experiments were performed employing DRM (caveolin-1 (cav-1)) and non-DRM (CD71) markers. Moreover, the cell surface receptor for cholera toxin, the glycolipid GM1, was detected with fluorescent labelled subunits of the cholera toxin, which represents a standard method for DRM identification [17]. Cav-1 is a major component of caveolae, which are flask-shaped invaginations of the plasma membrane involved in endocytic processes. Cav-1 is also present in lipid rafts, which are thought to be precursors of caveolae ("pre-caveolae") [18]. The transferrin receptor (CD71) is localized in clathrin coated pits or in other plasma membrane regions, but is absent from DRMs [17,19].

**Figure 2:**
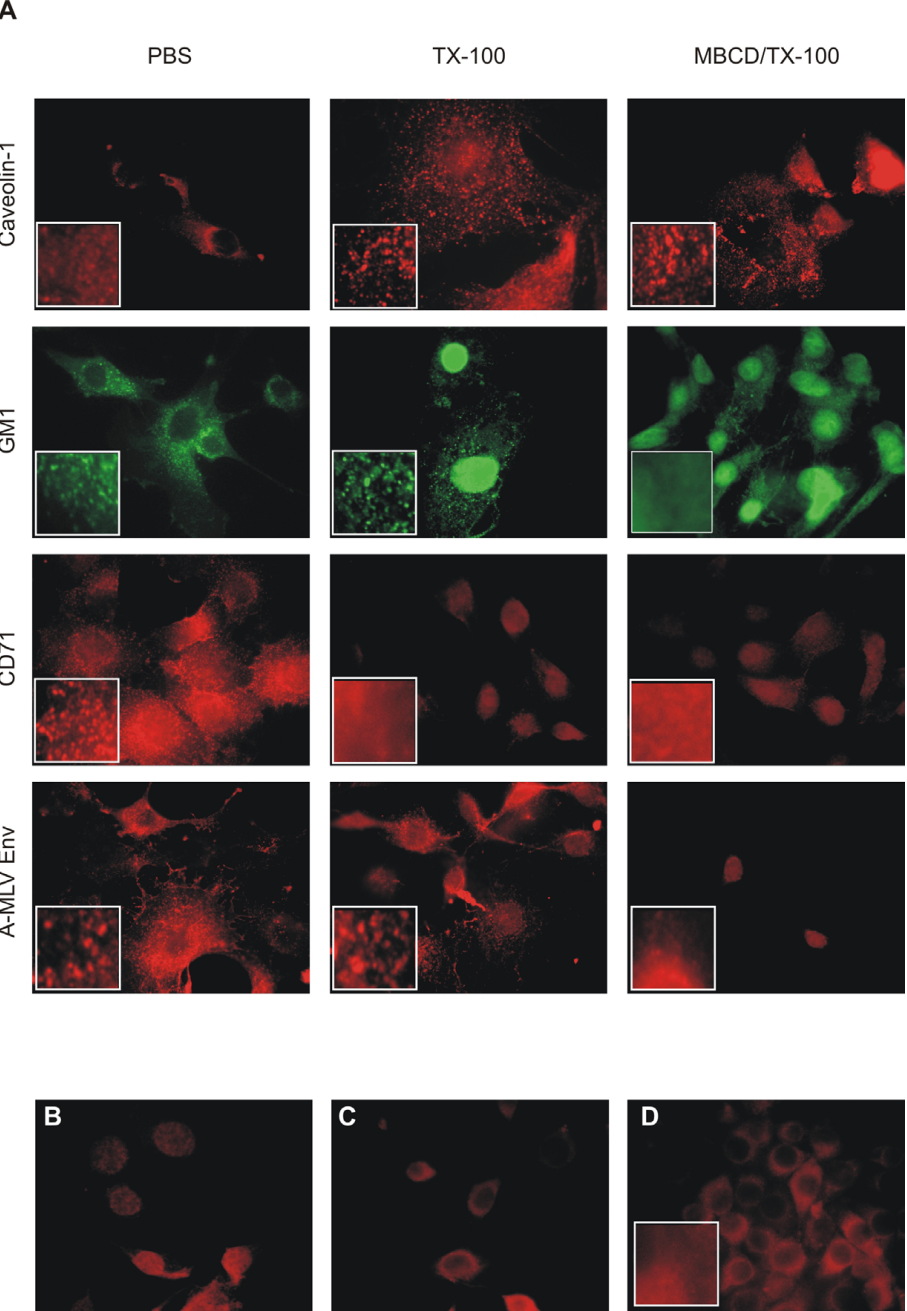
**Immunocytochemical investigations of the association of proteins with DRMs. A) **NIH3T3 cells producing A-MLV were treated with PBS, TX-100 or MBCD as indicated and subsequently subjected to TX-100 extraction and stained for cav-1, GM1, CD71 and A-MLV Env as indicated. **B) **Background of the secondary antibody used for cav-1 staining. **C) **Background of the secondary antibody used for A-MLV Env staining. **D) **NIH3T3 cells (Env negative) stained for A-MLV Env, negative control (see text for details). Photographs were taken using an oil immersion objective, original magnification 1000×.

Wild-type A-MLV releasing NIH3T3 cells grown on chamber slides were washed with PBS or 0.5% TX-100 at 4°C and subsequently fixed to the glass surface with paraformaldehyde. The cells were treated with filipin, a cholesterol-binding fluorescent dye [20], and stained for the DRM markers GM1 and cav-1 using FITC labelled cholera toxin or anti cav-1 antibody, respectively, and for CD71 using an anti CD71 antibody. A-MLV Env was detected using an anti-Env antibody (83A25 [21]). The relatively mild TX-100 treatment was sufficient to disperse CD71, which is not associated with DRMs, over the plasma membrane while the DRM markers GM1 and cav-1 as well as A-MLV Env remained as discrete spots (Fig 2A, compare left and middle columns).

In another set-up, the cells were first treated for 30 min with 5 mM methyl-beta-cyclodextrin (MBCD) at 37°C and subsequently with 0.5% TX-100 at 4°C prior to paraformaldehyde fixation and immunocytochemical staining (Fig. 2A, right column). MDCB is known to extract cholesterol from plasma membranes and is widely used to disrupt DRMs [22]. Enzymatic cholesterol determination revealed that approximately 60% of the cholesterol was removed from the plasma membrane upon MBCD treatment (data not shown). Due to disruption of the DRM structure, a combined MBCD/TX-100 treatment should result in dispersal of DRMs and proteins concentrated therein. Indeed, the combined MBCD/TX-100 treatment resulted in even distribution of GM1 as well as A-MLV Env fluorescence in the plasma membrane while cav-1 still was detectable in discrete spots in MBCD/TX-100 treated cells (Fig. 2A, right column). With respect to the distribution in the plasma membrane, TX-100 resistance, and MBCD extraction, A-MLV Env exhibits similar properties as the DRM marker GM1 and distinct properties compared to CD71. These findings are in agreement with the results obtained from density gradient centrifugations showing that A-MLV Env to a high degree is associated with DRMs.

### A-MLV Env co-localizes with DRM markers

**Figure 3:**
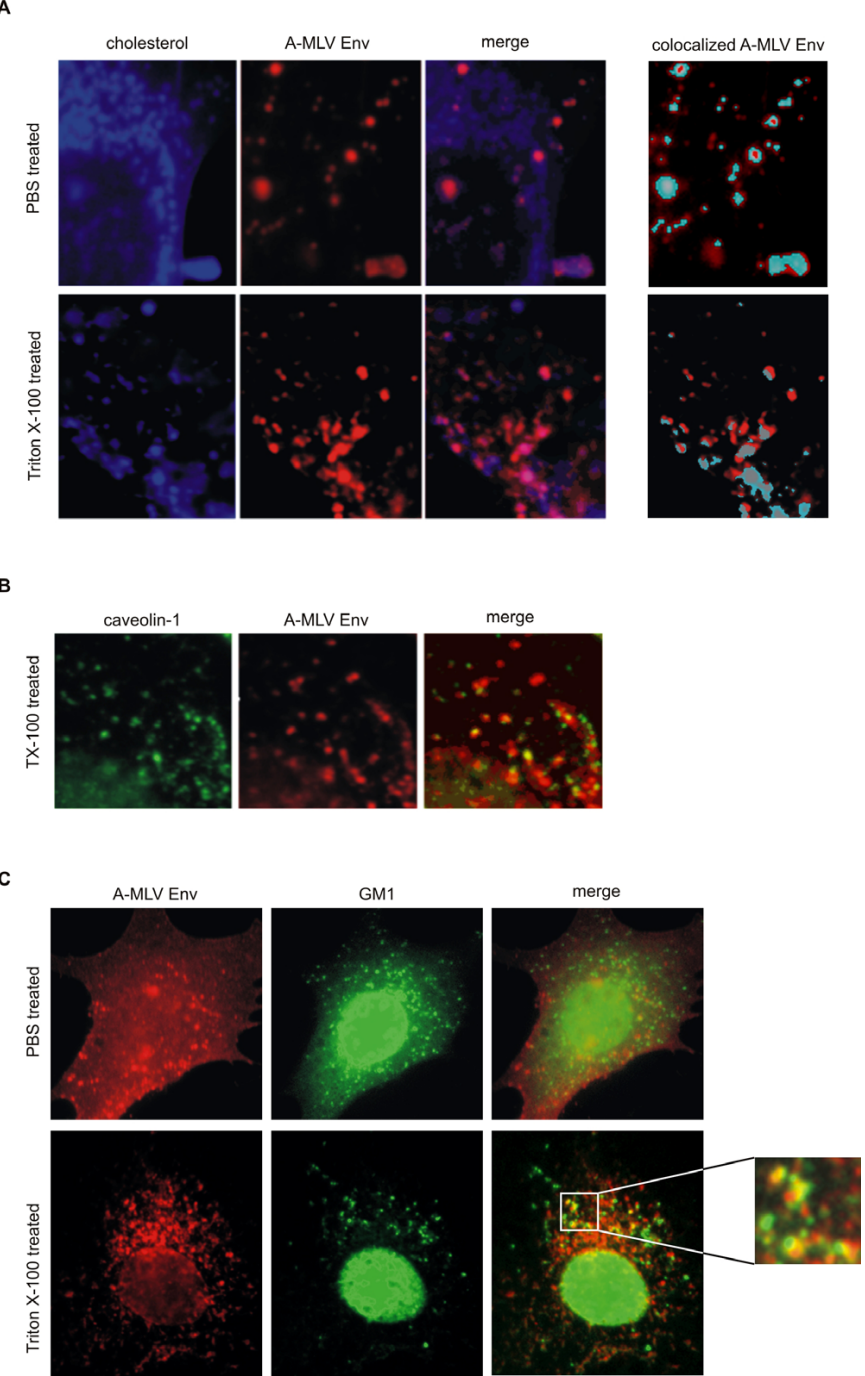
**A-MLV Env co-localization with cholesterol, GM1 and cav-1. A) **A-MLV Env co-localization with cholesterol. NIH3T3 cells producing wild-type A-MLV were treated with filipin for cholesterol detection (left column) and with an A-MLV Env specific antibody (second column) after fixation and treatment with PBS (top) or TX-100 at 4°C (bottom). Co-localization result in pink spots (merged images, third column). The column on the right shows the result of the co-localization finder plugin of the ImageJ program [30] merged with the original A-MLV Env staining. Turquoise colour indicates co-localization of A-MLV Env with cholesterol. **B) **A-MLV Env and cav-1 co-localization monitored by fluorescence microscopy. Immunofluorescent detection of cav-1 (left) and the A-MLV Env (middle) after treatment with TX-100 at 4°C in NIH3T3 cells producing A-MLV. Co-localization result in yellow spots (right). **C) **A-MLV Env (left) and GM1 (middle) were detected by immunofluorescence in A-MLV producing NIH3T3 cells after PBS (top) or TX-100 treatment at 4°C (bottom). Co-localization result in yellow spots (right). All photographs were taken using a fluorescence microscope and oil immersion objective, original magnification 1000×.

Finally, we performed co-localization studies of Env proteins with DRM markers in immunocytochemical experiments. Again, we used a combination of TX-100 treatment and immunocytochemical stainings. Wild-type A-MLV producing NIH3T3 cells grown on chamber slides were washed with PBS or 0.5% TX-100 at 4°C and subsequently fixed to the glass surface by paraformaldehyde treatment. The cells were incubated with filipin, a cholesterol-binding fluorescent dye [20], and DRM markers GM1 and cav-1 were detected using FITC labelled cholera toxin or anti cav-1 antibody, respectively. A-MLV Env was detected using an anti-Env antibody (83A25 [21]). As expected for a DRM-associated protein and from the results of the Dot Blot analysis (Fig. 1B and 1C), approximately 50% of A-MLV Env co-localized with cholesterol-rich spots (Fig. 3A). In accordance with the experiment shown in figure 2A, A-MLV Env did not disperse in the plasma membrane after TX-100 treatment (Fig. 3A). In addition, A-MLV Env also co-localized with cav-1 and GM1 resulting in yellow spots in merged photographs (Fig. 3B and 3C). No co-localization was observed when cells were stained for A-MLV Env and the non-DRM marker CD71 (data not shown).

Taken together, the immunocytochemical data confirm that of A-MLV Env to a large extent is associated with DRMs.

## Discussion

A number of previous investigations have shown that the plasma membrane of animal cells is a heterogeneous lipid bilayer that contains distinct cholesterol-rich micro-domains like DRMs, which are responsible for a number of biological functions e.g., concentrating and sorting of proteins [1]. A variety of viruses like HIV-1 and measles virus exploit DRMs for their assembly and budding [6,23] after association of certain structural proteins with DRMs.

Here we show that the major portion of plasma membrane A-MLV Env is associated with DRMs. Using biochemical and immunocytochemical methods we found that approximately 60–80% of A-MLV Env is localized in these microdomains. Similarly, Li et al. have reported that the closely related envelope protein of the Moloney murine leukaemia virus (MoMLV), which shows 62% identity to A-MLV Env on the protein level [2,24], is associated with rafts. Similar to MoMLV Env, A-MLV Env is not completely localized within DRMs. This is not uncommon for DRM-associated proteins as it has been shown for, e.g., HIV-1 p17 and gp41 [6].

The immunocytochemical method used here for investigation of the DRM association of A-MLV Env was shown to be suitable. The markers for DRM (cav-1, GM1) and non-DRM regions (CD71) of the plasma membrane exhibited the properties expected when the cells were treated with the non-ionic detergent TX-100. These experiments showed that A-MLV Env resembles GM1 or cav-1 upon treatment with TX-100. MBCD is known to dissolve DRMs by extracting cholesterol from the plasma membrane. As expected for a DRM associated protein, cholesterol extraction and subsequent treatment of the cells with TX-100 dispersed GM1 and A-MLV Env spots at the plasma membrane. In contrast, cav-1-positive spots were still detectable even when these were depleted of cholesterol (data not shown). This is in accordance with a previous investigation demonstrating that only a negligible amount of cav-1 could be released through MBCD treatment [22]. Probably, MBCD resistance of caveolin-spots is due to the fact that the caveolin proteins build up a close network on the luminal side of the plasma membrane [25]. Furthermore, A-MLV Env co-localizes with the DRM markers cholesterol, cav-1 and GM1 confirming that A-MLV Env to a high degree is associated with DRMs.

Retrovirus assembly and release is solely driven by the viral Gag polyprotein [28], thus virus-like particles are formed in the absence of any other viral proteins or genome. Since, the spatial neighbourhood of Env and Gag proteins is a prerequisite for release of functional viral particles, the localisation of A-MLV Env within DRMs may be indicative of viral budding from these regions. This model is supported by the fact that a 1.4 fold increase of the cholesterol content of the plasma membrane of NIH3T3 cells resulted in a more than 3-fold increase of viral membrane cholesterol of amphotropic MLV (A-MLV) released from these cells [14].

Our finding may have consequences for the understanding of A-MLV assembly and budding, which is known to be a specific and coordinated process. In the case of A-MLV, previous data indicated that the viral components assemble and bud at the cellular plasma membrane (reviewed in [8]). Recent investigations of Sandrin et al., however, demonstrate intracellular co-localization of A-MLV Env and MoMLV core proteins in the endocytic pathway in late endosomes including multivesicular bodies (MVBs). They suggest that the interaction of MLV Env and core proteins in these compartments could influence virus particle formation [27]. According to general belief DRM like microdomains are already formed in the Golgi, and it is thus possible that A-MLV Env and core proteins are already sorted intracellularly in the same compartment and transported together to the plasma membrane.

Co-localization has been suggested to be sufficient for incorporation of cellular proteins into virions [26]. Since cav-1 and A-MLV Env co-localize in mouse NIH3T3 cells the putative presence of cav-1 in A-MLV virions would indicate that A-MLV buds from cav-1 containing DRMs. Interestingly, we have found that cav-1 is incorporated into A-MLV virions, whereas no CD71 could be detected (Beer and Wirth, unpublished data). However, whether cav-1 plays a specific role in viral protein sorting to the plasma membrane and viral assembly is presently not known, but this issue is subject of current investigations.

Nevertheless, based on the specific properties of individual DRMs, like rafts or caveolae, rafts seem to be most suitable for virus assembly and budding. The invagination of caveolae within the plasma membrane of the cells, their involvement in endocytic processes and, moreover, their compact coat of caveolin-oligomeres [25] presumably exclude caveolae as suitable regions for viral budding and suggest rafts as budding platforms for A-MLV.

## Conclusions

Taken together, our findings provide evidence that A-MLV Env is localized in DRMs, similar to the Env of the closely related E-MLV [2,26] and lentiviral HIV-1 Env [6] These results suggest that rafts are budding platforms for A-MLV in NIH3T3 and 293T cells.

## Methods

### Cells

NIH3T3 (ATCC CRL-1658) and 293T (ATCC CRL-11268) cells were propagated in DMEM supplemented with glutamine and 10% FCS. Antibody producing hybridoma cells were grown in RPMI 1640 medium supplemented with glutamine and 1% ultra low IgG FCS (Gibco). All cells were grown at 37°C, 5% CO2 and 95% humidity.

### Plasmids, transfection and helper virus approach

pMLVampho contains the complete genome of A-MLV cloned into pBluescript (Genethon, France received via J.-C. Pages). A-MLV producing NIH3T3 cells resulted from transfection of pMLVampho [29] and a subsequent infection of NIH3T3 cells with replication-competent MLV-A.

### Antibodies and antibody production

Hybridoma cell lines were used for the production of rat monoclonal immunoglobulin G (IgG) antibodies against MLV SU (83A25, kindly provided by L.H.Evans [21]). To concentrate the antibodies, the cell suspension was centrifuged at 2000 × g for 10 minutes. 29.1 g ammoniumsulfat per 100 ml were added and the supernatant stirred for 1 hour at 4°C. After centrifugation (27000 × g, 4°C, 1 h), the pellet was resuspended in PBS and the antibody solution dialyzed against PBS. For Western and dot blot analysis rabbit anti rat IgG coupled to horseradish peroxidase (HRP) (Sigma) was used. Antibody to mouse CD71 was purchased from ebioscience and to caveolin from BD Bioscience. Fluorescein isothiocyanate (FITC)-conjugated goat anti rat IgG was obtained from Sigma and Texas Red labelled goat anti rabbit IgG was purchased from Calbiochem. Texas Red conjugated goat anti rat IgG and FITC-conjugated goat anti rabbit IgG were obtained from Jackson Immunoresearch.

### Triton X-100 extraction and sucrose gradient

To investigate the association of the A-MLV envelope protein with cholesterol-rich microdomains, 293T cells were transfected with either pEGFP-N1 (Clontech) or A-MLV Env encoding plasmids [15] using the calcium phosphate precipitation method. 48 hours after transfection, the cells were washed with 1×PBS, overlaid with 1×PBS and washed from the cell culture flask surface. The cells were pelleted with 300×g at 4°C and resuspended in icecold 1×PBS containing 1% TritonX-100 and 1 mM Pefabloc (Sigma). The cells were incubated 30 min on ice and adjusted to 40% sucrose or OptiPrep and loaded into SW60Ti-tubes. The samples were overlaid with a discontinuous sucrose or OptiPrep gradient (35% – 5%). The gradient was centrifuged at 4°C with 40000 rpm for 20 h in a SW60Ti rotor. Six fractions were collected from the top of the tube.

An equal volume of acetone was added to the fraction and incubated at -20°C. The precipitated proteins were pelleted by centrifugation and dried at room temperature. The pellet was resuspended in 1×SDS gel loading buffer. The fractions were analysed for their egfp-N1 or A-MLV Env protein content using a 12% SDS gel and Western Blot. Anti-gfp antibody was obtained from Abcam (AB290). A-MLV Env was detected using antibodies produced by the hybridoma cell line 83A25.

### Dot immunoassay

To investigate the association of proteins with cholesterol-rich microdomains via Western or Dot Blot the extraction of TX-100 soluble proteins was performed as described previously [6] with the following modifications. NIH3T3 cells were washed with PBS, overlaid with 4°C cold 0.5% Triton X-100 in the presence of a protease inhibitor cocktail (Pefabloc, Sigma) and gently shaked at 4°C for 1 min. The supernatant was removed and stored on ice. The remaining cells were suspended in PBS and homogenized in a RiboLayser tube at 6000 rpm. The stored soluble protein fraction was adjusted to 40% sucrose in TKM buffer (50 mM Tris-HCl, pH 7.4; 25 mM KCl; 5 mM MgCl_2_; 1 mM EDTA) and loaded into SW40Ti-tubes. The sample was overlaid with 35% to 5% sucrose (5% steps). The gradient was overlaid with the homogenized cell pellet. The gradient was centrifuged at 4°C with 38000 rpm for 20 h. Six fractions were collected from the top of the tube. 100 μl portions of each fraction were diluted with 400 μl PBS, filled into the wells of a Bio Dot apparatus (BioRad) and gently suctioned onto nitrocellulose membranes (MilliPore). The membrane strips were blocked for 1 h with Tris-buffered saline containing 10% horse serum and 3% bovine serum albumin. To detect A-MLV envelope and cav-1 proteins the membrane was incubated over night with antibodies against the proteins in blocking buffer at a 1:200 (Env) and 1:5000 (cav-1) dilution. The secondary antibodies, rabbit anti rat and goat anti rabbit coupled to HRP, were used at a 1:1000 dilution. The dot blots were developed with TMB stabilized substrate for HRP (Promega). The spot intensities were quantified using Easy Win 32 (Herolab).

### MBCD treatment

To extract cholesterol out of the cellular plasma membrane NIH3T3 cells were overlaid with 5 mM Methyl-β-cyclodextrin (MBCD, Sigma). After slightly shaking at 37°C for 30 min, the cells were used for further treatment with Triton X-100.

### Immunofluorescent staining

NIH3T3 cells were seeded onto chamber slides (Nunc) and grown to 80% confluency. After washing once with PBS, the cells were overlaid with 200 μl PBS or 0.5% Triton X-100 (4°C) and incubated for 1 minute at 4°C (gently shaking at 8 rpm). Afterwards the cells were immediately overlaid with 4% paraformaldehyde and incubated for 15 min at RT. After washing with PBS and blocking with Tris-buffered saline containing 10% horse serum and 3% bovine serum albumin antibodies against A-MLV Env, and cav-1 were added. The cells were overlaid with secondary antibodies after washing with PBS. After a final washing step with PBS the slides were mounted with immunofluorescence mounting medium (Dako).

For co-localization studies the cells were blocked a second time after incubation with the secondary antibody and stained for GM1 with FITC-conjugated cholera toxin (Calbiochem, 8 μg/ml), for cholesterol with filipin (Sigma, 50 μg/ml) or cav-1 as described above.

A fluorescence microscope (Axiovert TV135, Zeiss; filter sets: filipin – XF113, 387/450 nm (Em/Ex), FITC – 495/520 nm, Texas Red – 595/615 nm; Omega filters) at 1000× magnification was used for the detection of the stained proteins. Images were taken using a cooled CCD camera (PXL 1400, Photometrics), digitalized, pseudo-coloured and merged (IPLab Spectrum). Brightness and contrast were adjusted.

## Competing interests

The author(s) declare that they have no competing interests.

## Authors' contributions

CB conceived of the study, carried out the experimental work and helped to draft the manuscript. MW participated in the design of the study, supervision of conduction of the experiments and drafted the manuscript. LP helped with coordination and design of the density gradients. All authors read and approved the final manuscript.
